# Developing a Model for Quantifying QTc-Prolongation Risk to Enhance Medication Safety Assessment: A Retrospective Analysis

**DOI:** 10.3390/jpm14020172

**Published:** 2024-01-31

**Authors:** Luis Giovannoni, Gerd A. Kullak-Ublick, Alexander Jetter

**Affiliations:** 1 Department of Clinical Pharmacology and Toxicology, University Hospital Zurich, University of Zurich, Rämistrasse 100, 8091 Zurich, Switzerland; 2 Tox Info Suisse, National Poison Center, Associated Institute of the University of Zurich, Freiestrasse 16, 8032 Zurich, Switzerland

**Keywords:** QTc prolongation, QT-prolonging drugs, medication safety assessment

## Abstract

There are currently no established methods to predict quantitatively whether the start of a drug with the potential to prolong the QTc interval poses patients at risk for relevant QTc prolongation. Therefore, this retrospective study aimed to pave the way for the development of models for estimating QTc prolongation in patients newly exposed to medications with QTc-prolonging potential. Data of patients with a documented QTc prolongation after initiation of a QTc-prolonging drug were extracted from hospital charts. Using a standard model-building approach, general linear mixed models were identified as the best models for predicting both the extent of QTc prolongation and its absolute value after the start of a QTc-time-prolonging drug. The cohort consisted of 107 adults with a mean age of 64.2 years. Patients were taking an average of 2.4 drugs associated with QTc prolongation, with amiodarone, propofol, pipamperone, ondansetron, and mirtazapine being the most frequently involved. There was a significant but weak correlation between measured and predicted absolute QTc values under medication (r^2^ = 0.262, *p* < 0.05), as well as for QTc prolongation (r^2^ = 0.238, *p* < 0.05). As the developed models are based on a relatively small number of subjects, further research is necessary to ensure their applicability and reliability in real-world scenarios. Overall, this research contributes to the understanding of QTc prolongation and its association with medications, providing insight into the development of predictive models. With improvements, these models could potentially aid healthcare professionals in assessing the risk of QTc prolongation before adding a new drug and in making informed decisions in clinical settings.

## 1. Introduction

Measurement of the QT interval and its adjustment for heart rate is a matter of great importance to physicians, drug manufacturers, and regulatory agencies because of the relationship between prolongation of the QT interval and potentially lethal ventricular arrhythmias, such as torsades-de-pointes (TdP), which may degenerate into ventricular fibrillation and result in sudden cardiac arrest [1,2]. The length of the QT interval correlates with heart rate (QTc), with a proportional decrease as the pulse increases; therefore, the QT interval should always be corrected for the actual heart rate, where the normal range of QTc is below 450 ms in males and 470–480 ms in females [3].

Numerous risk factors have been identified that prolong the QT interval, including increasing age, female sex, genetic variants, cardiovascular diseases, and electrolyte disturbances [3]. However, the most common culprits are those associated with QT-prolonging drugs, which are still regularly used in clinical practice, namely antiarrhythmics, antihistamines, antibiotics, antidepressants, neuroleptics, and prokinetics [4]. When drugs that can prolong the QT interval are used, physicians should ensure that the potential benefits are clinically important and the risks are minimized. However, with many drugs that can cause QT interval prolongation, the risk of TdP is so low that most experts do not consider measurement of the QTc interval to be cost-effective [5]. It nevertheless must be said that approximately 7.1% of the patients admitted to an acute unit had a prolonged QTc interval [6] and that for every 10 ms increase in a prolonged QTc, there is another 5% increased risk of arrhythmic events [7].

These considerations reinforce the need for a mathematical model that can be applied to each patient in an individualized manner and that can lead to a prediction of the QTc interval under determinate conditions. Thereby, it could help make the decision to withhold prescribing first-line therapies in a given situation more rationally, as the resulting risk would be excessively increased. The prescriber may then consider whether the specific high-risk medication is nevertheless needed or whether an alternative drug with a lower risk of QTc interval prolongation could be substituted [2]. Similarly, in a multitude of situations where acting overly cautious leads to forgoing the prescription of a particular therapy, a more detailed knowledge of the repercussions of that drug on the QTc interval could help in basing a decision on personalized and more detailed arguments. In patients with no risk factors for QTc prolongation, the risk of withholding first-line therapies is major when compared with drug-induced TdP, resulting in a higher risk of adverse outcomes [8].

Many clinical decision support systems generate alerts when QT-prolonging drugs with a known risk of TdP are combined. Berger et al. reported that more than 40% of processed drug prescriptions lead to drug safety alerts [9]. In a literature review in 2006 analyzing 17 papers, van der Sijs et al. found that drug safety alerts are overridden by clinicians in 49% to 96% of cases [10]. As a distinction between appropriate and useful alerts should be made, patient-tailored alerts based on a detailed model to estimate the QTc interval prolongation could increase the specificity of the alerts and so reduce overriding [10].

Over the recent years, emerging consensus is growing about personalized and precision medicine, a targeted treatment based on individual patient characteristics [11]. Better strategies to educate and train healthcare professionals about personalized medicine must be developed and implemented [12]. Until now, only a few other studies have tried to predict QTc prolongation with statistical models, using clinical and routine laboratory data. In 2018, Bindraban et al. developed and validated a risk model for the prediction of QT interval prolongation [8]. The authors aimed to implement the risk model in a clinical decision support system, supporting the management of the risks involved with QTc-interval-prolonging drugs [8]. They conclude that implementing a clinical decision support system in medication surveillance can reduce the number of alerts for patients with a low risk of QTc prolongation [8].

Before starting a QT-prolonging drug, patients should be assessed for risk factors for QT prolongation and an assessment of the risk/benefit balance of initiating the QT-prolonging drug should be carefully made [13]: the risk of ventricular arrhythmias under medication should be compared with the morbidity and mortality of the untreated underlying condition. It goes without saying that every case requires a peculiar evaluation: for example, the benefit of an antiarrhythmic therapy with sotalol is best when it results in the immediate termination of a sustained ventricular arrhythmia [5,14], but when the same therapy is used to treat chronic arrhythmias, the risk may outweigh the benefit [5].

In this context of relative uncertainty and speculation, the introduction of a model to predict QTc prolongation could assist practitioners who face the dilemma of whether to prescribe a medication—even after correcting modifiable risk factors for QT prolongation [13]—or renounce, aware that the therapy would do more harm than good. At present, some models to predict the individual hazard of QTc prolongation—based on easily obtainable clinical risk factors [2]—have been developed, but only a few of them can estimate the extent of QTc prolongation caused by a drug newly added to an existing regimen.

Therefore, the aim of this study is to outline a model to predict quantitatively the extent of QTc prolongation and the absolute QTc time, respectively, when a QTc-prolonging drug is newly begun. This objective necessitates the identification of parameters of influence on these two outcome variables, the development of suitable models for prediction, and in principle also the external validation of the models developed. While the first two objectives could be investigated here, the external model validation could not be achieved due to the lack of suitable additional data, although it was not a specific objective at this stage.

## 2. Materials and Methods

### 2.1. Study Design and Settings

This retrospective study was conducted at the University Hospital of Zurich (USZ) consulting an internal database on in-hospital adverse drug reactions (ADRs), managed by the local Department of Clinical Pharmacology and Toxicology. Selection criteria were essentially three: the notification should report on a documented QTc prolongation after initiation of a QTc-prolonging drug, which occurred between 1 January 2015 and 31 December 2020, and the involved patients were requested to have endorsed the general consent for medical research. Data were extracted retrospectively using the USZ patient record database KISIM (Cistec AG, Zurich, Switzerland). The study was approved by the Ethics Committee of the Canton of Zurich (BASEC-Nr. 2019-02123) and carried out respecting all pertinent laws and regulations in force in Switzerland at the time of data collection [15].

The following data were collected consulting the computerized medical records of the selected patients as far as available: demographic data, previous medical history and diagnoses, laboratory data, current and past medications, and ECGs. In the first phase, ECGs with a documented QTc prolongation in a close timely correlation with the initiation of a new medication cataloged on the CredibleMeds^®^ (AZCERT, Tucson, AZ, USA) [16] list of drugs with a known and a possible risk associated with QTc prolongation were identified.

Subsequently, an attempt to identify an electrocardiographic measurement of the QTc before initiating the medication and after terminating the therapy was made. In a complementary manner, demographic, clinical, and laboratory information were extracted. This collection was restricted to proven risk factors for QTc prolongation, according to the existing literature [3,17]. Clinical information was extracted by consulting relevant medical, anesthetic, and surgical reports, daily progress notes, administration schedules, and dose regimens.

For purposes of assessing the QTc interval prolongation risk associated with serum potassium, magnesium, calcium, and creatinine concentrations, values recorded closest to the time that QTc interval prolongation was initially documented were used [2]. Serum magnesium, calcium, and TSH concentrations were available for some, but not all patients, as they are not routinely ordered for every patient. Creatinine clearances were calculated from serum creatinine concentrations using the CKD-EPI (Chronic Kidney Disease Epidemiology Collaboration) equation [18]. The category of “antihypertensives” includes angiotensin II receptor blockers (ARBs), ACE inhibitors, and calcium channel blockers (CCB).

Regarding the CredibleMeds^®^ drugs, data on the intake period and the dosage in order to assess a possible temporal and quantitative relationship with the QTc prolongation were collected. Due to the small number of patients considered for the definitive analyses, the original intent to develop a model that contained specific drugs as predictors could not be fully achieved: for this reason, the focus was set merely on the potential relationship between the initiation of a new QT-prolonging medication and the onset of a QTc prolongation. QT intervals were corrected for heart rate automatically using Bazett’s formula (QTc). QTc interval prolongation was defined as a QTc interval ≥ 450 ms for male patients and ≥480 ms for females [2,3,4]. Selected patients had a QTc prolongation according to this definition at the time of taking the considered drugs. For patients with an already elongated QTc before medication intake, further prolongation was considered for the analyses. In contrast, patients were not included if they had an elongation during drug treatment leading to a QTc below the above-mentioned values. As the full extent of personal variability of the QT interval is currently unknown, the use of specific QT interval cut-off values must be interpreted in the context of specific clinical information [19]. Comparing existing literature and the experience based on clinical practice, a QT prolongation was considered as described above.

### 2.2. Patient Population

**Figure 1:**
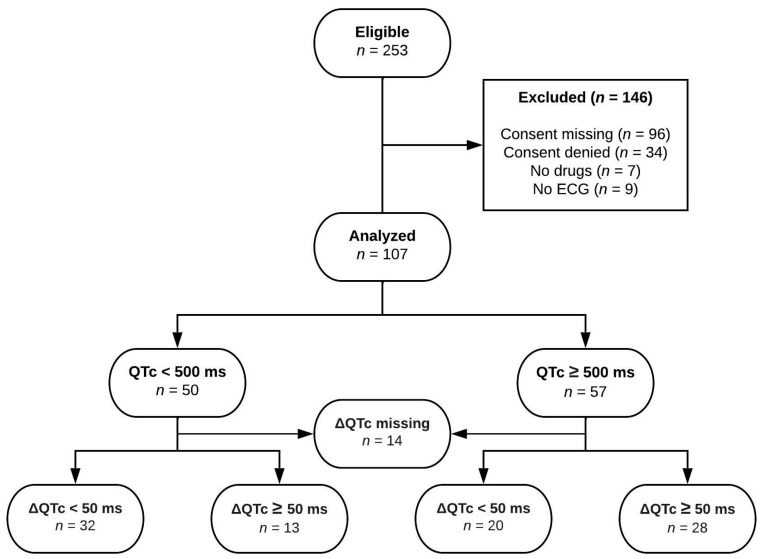
Study flow diagram. QTc indicates absolute QTc time under medication and ΔQTc the QTc prolongation compared to baseline QTc.

A total of 253 eligible patients were screened for the study. However, only 107 (42.3%) of them were actually considered in the analysis, as an important portion of the patients could not be analyzed because there was no information regarding explicit consent or because explicit consent had been denied a priori (Figure 1).

### 2.3. Design of the Models

Statistical analyses were performed using IBM SPSS Statistics (IBM, Amonk, NY, USA, Version 28). For the used continuous predictors, frequencies, mean, and standard deviation (σ) were calculated and displayed if normally distributed, while median and IQR were used in the case of not normally distributed values or very small samples. For categorical binary predictors, frequencies obtained from the variable’s frequency distribution table were assessed.

The assumption of normality in the distribution of continuous predictors used in the analyses was never consistently violated (tested by visual assessment with boxplot diagrams and with common tests of normality). For this reason, it was not necessary to transform data to their natural logarithm, as the used statistical models are robust to violations of normality [20]. Also, the dependent variables absolute QTc under medication and QTc prolongation were essentially normally distributed, as assessed by histograms and Q-Q-diagrams.

To assess the relationship of every single independent variable on the dependent variable—namely the QTc prolongation and the absolute QTc value during treatment, respectively—Student’s *t*-tests, simple ANOVA tests, and correlation analyses were performed (Supplementary Tables S1 and S2). For the multivariate analysis, the parameters based on the following criteria were selected. First, the variables with the best significance in the univariate analysis were considered, initially accepting—considering the small sample size—even variables with a *p* > 0.05, and if physiological plausibility was present. Second, data that led to an important reduction of the dimensionality of the dataset and that were clearly not compatible with current knowledge of physiology were excluded [15].

Then, the identified parameters were used as constituents of a more comprehensive model, with the aim of reproducing data collected in clinical practice. Therefore, different mathematical models using a backward stepwise selection were built, which were then weighted up by comparing the statistical significance (α) of the single components and using the Akaike information criterion (AIC) as a means for the model selection [21]. Generalized linear mixed models, general linear models, and linear mixed models were used.

To achieve the most accurate prediction of the prolongation of QTc and its absolute value, the general linear mixed model (GLMM) was used, based on the following equation [22]:

*n_ij_* = *β_0j_* + *β_1j_x_1ij_* + … + *β_Pj_x_Pij_*

where *n_ij_* is the searched predictor, *β_0j_* is a model-specific random intercept, and *x_pij_* (*p* = 0, …, *P*) are level-1 covariates with fixed regression coefficients *β_p_.*

Two-sided tests were considered significant when the *p*-value was <0.05. The accuracy and clinical applicability of the statistical models were reviewed by comparing the measured and predicted endpoints of all observations using Pearson’s correlation [15]. Regarding the final models, two variables with a *p*-value > 0.1 (potassium and loop diuretics) were included in both models because both factors in question constitute a significant risk element from the biological point of view, as also reflected by the coefficients in the model.

## 3. Results

### 3.1. Patients

In total, 253 patients were potentially eligible for the study. A total of 146 patients (58%) were excluded because of violations of the inclusion criteria (Figure 1). Hence, 107 patients with a documented QTc prolongation were included in the investigation. The mean age of the patients was 64.2 years, 68 (63.6%) patients were men, and 39 (36.4%) were women. The mean QTc before the start of the QTc-prolonging drug amounted to 445.2 ms and was assessed in 93 (87%) participants. The mean QTc value after the start with a drug with known QTc-prolonging potential was 497.7 ms, with a mean increase (ΔQTc) of 50.7 ms.

The majority of patients were inpatients not treated in intensive care units (61.7%), affected by hypertension (68.2%), and treated with loop diuretics (60.7%). Other common medical conditions were also represented, but less frequently: 26 patients (24.3%) had ischemic heart disease, 24 (22.4%) were diagnosed with chronic heart failure, 17 (15.9%) suffered from diabetes mellitus, and 44 (41.1%) had cardiac arrhythmia. All included patients were taking at least one medication. Patients were taking a median of 2.4 drugs listed in the CredibleMeds^®^ database, known to be associated with both QTc prolongation and torsades-de-pointes. As common knowledge, all antiarrhythmic drugs have the potential to provoke arrhythmias [23], and a remarkable percentage of participants were treated with antiarrhythmic drugs (31.8%). Moreover, the risk of arrhythmia is increased in patients with abnormal cardiac substrate, with electrolyte abnormalities, and during drug initiation [23]. Presumably because of this increased risk, patients with a structural cardiac abnormality are overrepresented in the population analyzed for this study. Table 1 and Table 2 present statistics of the investigated population.

As previously outlined, patients were selected if a QTc prolongation occurred after starting treatment with a drug included in the CredibleMeds^®^ list. The five most frequently involved drugs were amiodarone in 34 patients (31.8%), propofol in 32 patients (29.9%), pipamperone in 27 patients (25.2%), ondansetron in 22 patients (20.6%), and mirtazapine in 17 patients (15.9%). Most patients were taking more than one drug associated with QTc prolongation (71.0%), with a maximum of seven drugs within one single patient, and 99 patients (92.5%) taking between one and four drugs.

### 3.2. Final Models

The different regression coefficients of the predictors constituting the model estimating the absolute value of the QTc under medication are displayed in Table 3.

Equation (1) summarizes the proposed model, showing how the dependent variable can be predicted by the independent variables identified and included in the model above. For categorical variables, if the predictor is present, its coefficient is included in the formula by multiplying it by one. If it is absent, multiplication by zero eliminates the variable. In the case of quantitative variables, the coefficient is multiplied by the value of the variable.

QTc under medication = 444.2 − 0.348 × age + 0.228 × initial QTc + 10.652 × diabetes mellitus + 14.251 × ischemic cardiomyopathy + 9.48 × arrhythmia + 6.671 × loop diuretics(1)

Similarly, a model was constructed for QTc prolongation (ΔQTc), the coefficients are shown in the table below (Table 4). The corresponding Equation (2) is displayed underneath.

QTc prolongation = 111.7 − 5.591 × [K^+^] + 11.596 × antihypertensives − 0.307 × age + 20.668 × arrhythmia (2)

### 3.3. Correlation between Measured and Predicted Data

**Figure 2:**
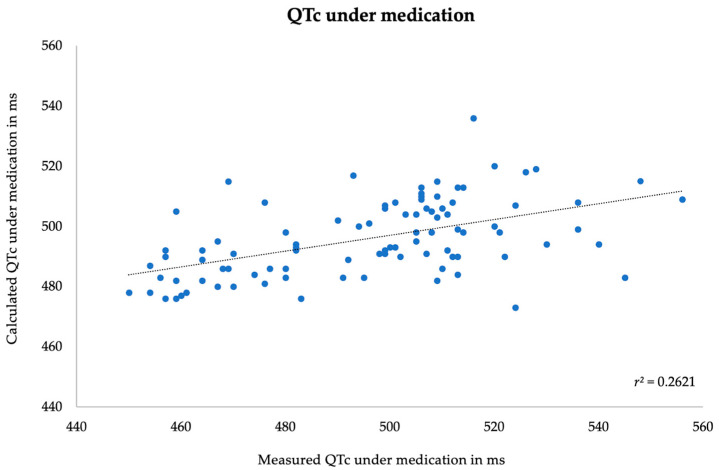
Correlation between measured and predicted absolute QTc value under medication.

**Figure 3:**
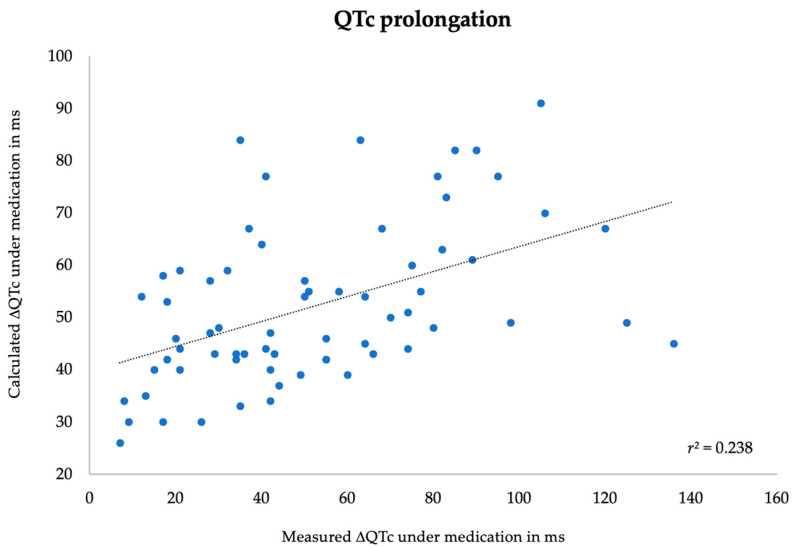
Correlation between measured and predicted QTc prolongation under medication.

Finally, the predicted value for every patient using each of the two models was calculated, comparing the obtained result with the measured value. In both models, the value of QTc under medication interval and its prolongation (ΔQTc) showed a significant correlation between measured and calculated values, although only with a weak strength of association. The Pearson’s correlation coefficients (r) of absolute QTc interval under medication and QTc prolongation (ΔQTc) were 0.51 and 0.38, respectively. The *p*-value was <0.001 in both analyses. Scatter plot diagrams are shown below in Figure 2 and Figure 3. The external validity of the models has not been assessed.

## 4. Discussion

In this study, clinical data were collected in a population with documented QTc prolongation after the initiation of a medication known to be associated with this potentially dangerous adverse drug reaction. This study showed that the development of a predictive model estimating the extent of QTc prolongation following the initiation of at least one drug associated with this side effect and starting from other known risk factors is feasible. It is important that particular attention be focused on the pharmacologic component, as QT-prolonging medications and electrolyte abnormalities have a greater effect on mortality rates than QT prolongation caused by other diagnoses [24].

In this work, the importance of drugs and polypharmacy in the context of acquired long QT syndrome (LQTS) became apparent again. Almost all patients—in addition to the drugs on the CredibleMeds^®^ list—were simultaneously taking other drugs. Several studies focused on the role of drug–drug interactions, CYP450 inhibition, and polypharmacy in QTc prolongation [3,4,17,25,26,27]. As LQTS is one of the critical diseases constituting an augmented risk for sudden cardiac death [28], developing a clinically applicable model to predict the extent of QTc prolongation could be helpful in preventing subsequent TdP, even more so considering that the existing literature agrees on the mortality rate of TdP with values ranging between 15 and 20% [29,30].

The fact that increasing age is associated with an increased risk of QTc prolongation [3] is also supported in this work. Although sex was not a relevant factor of influence in the developed prediction models for QTc time under medication or the QTc prolongation, respectively, it should be emphasized that earlier literature evidenced how sex hormones can influence the QTc duration by acting on ion channels within the heart cells [31]. For further analysis, it might be interesting to consider subpopulations to better investigate this aspect at various life stages, especially during pregnancy or in the peri- and postmenopausal periods [31].

Another recurring aspect in the presented models is the fact that patients with underlying cardiovascular diseases are particularly at risk for QTc prolongation [32]: physiological knowledge and the existing literature show that prolongation of the QTc interval could be promoted by myocardial and electrophysiological remodeling processes [33]. Myocardial changes induced by hypertensive cardiomyopathy and sympathovagal imbalance are the likely mechanisms behind the relationship between hypertension and QTc prolongation [34].

Similar to this work, the model developed by Bindraban et al. also included patients using one or more QT-prolonging drugs according to the CredibleMeds® list, including both inpatients and outpatients [8]. Despite some differences in methodology, several risk factors were identified which are also included in the models presented in the current study. In both works, hypokalemia, a previously documented QTc prolongation, and the use of loop diuretics were identified as risk factors. Differences between the models can probably be explained by the differences in size and composition of the study populations and in the variables studied [8].

It is well known that hypokalemia is a risk factor for the occurrence of TdP in patients with long QTc intervals. Both models include either potassium or loop diuretics—often ranked as one of the leading causes of hypokalemia—depending on which parameter fits better. Although not statistically significant, both parameters showed large coefficients influencing the respective equations and improving the models as assessed with the Akaike criterion. An important work by TeBay et al. focused on the role of the human Ether-à-go-go-related gene (hERG) in the acquired LQTS, a gene that encodes a potassium ion channel essential for cardiac repolarization [35]. The study in question not only revealed that potassium is an independent factor associated with a prolongation of the QTc interval but also demonstrated how it potentiates the prolonging effect of other drugs, such as quinine [35]. Loop diuretics are independently associated with LQTS [2]. Considering the current knowledge about hERG, they may have multiple implications in the pathways responsible for QTc prolongation.

The current study has limitations. First of all, the limited number of patients considered, caused by the absence of information about explicit consent for further use of their clinical data or its denial, constituted an important limitation to the quality of the extrapolated statistical data. In addition, the quality of the extrapolated data is difficult to verify, as a large part of the considered parameters comes from measurements performed in various settings and by different actors, contributing to a greater probability of running into errors. On the other hand, this may also be taken as a strength, because in everyday clinical routine, data quality is usually limited. However, it would have been interesting to have more data, particularly to better analyze the role of the ingested drugs individually. This would enable investigating whether the extent of QTc prolongation also depends on factors such as the substance, the administered quantity, and the route of administration. In any case, the aim of this work was to provide a starting point for subsequent research, likely contributing to the development of a more comprehensive and accurate model. This model should be validated in an independent dataset and might subsequently play a role in daily clinical practice.

Due to the limited number of patients in the study, external model validation with parallel group and subgroup analyses could not be conducted. The small number of datasets also resulted in some parameters, whose inclusion improved the model according to the Akaike criterion, not reaching statistical significance in the GLMM assessment. Similarly, the predictive power of the models was relatively weak, indicated by the low *r*^2^ values for the correlations between observed and predicted QTc times and prolongations, respectively. Another limitation could be that only inpatients were selected, automatically excluding many healthcare settings where QT prolongation occurs. Finally, the considerable variability in calcium concentrations, arising from the impossibility of correcting them systematically according to albuminemia due to insufficient information, led to the exclusion of this variable from the models. As the QTc interval is significantly prolonged in hypocalcemia and correlates with the extent of hypocalcemia, this parameter could constitute an important element within a future predictive model.

Further research on this important topic is needed, as the impact of QTc prediction on clinical practice is decisive, with a clear potential to improve patient care and risk assessment. The considerations articulated thus far strongly support how QTc prediction models can enable clinicians to safely prescribe medications to individuals at risk of QTc prolongation, a critical factor in drug-induced arrhythmias. This way is central to enhancing medication safety, guiding clinicians in personalized treatment decisions, reducing potential adverse effects, and improving outcomes.

One aspect that certainly warrants further exploration is the inclusion of single medications as variables in predictive models, rather than developing a single model for all QTc-prolonging drugs [4,36,37]. While such an approach would make the model more specific, it may reduce its practical utility. This aspect could not be developed further as a consequence of the small number of patients included. It can be estimated from the exhibited results that the total number of patients has to be much higher to adequately address this question [5,37].

## 5. Conclusions

In this exploratory study, two distinct models were developed, enabling the estimation of the extent of QTc prolongation and its absolute value in patients who will be exposed to at least one medication known to prolong the QTc interval. However, these predictions are so far imprecise and, hence, cannot be directly implemented into clinical practice. Further research is necessary to ameliorate and validate the proposed models, making them clinically applicable and reliable in daily practice.

## Figures and Tables

**Table 1:** Descriptive statistics of qualitative parameters of the study population used for statistical models.

Parameters			N
	Sex	male	68
		female	39
Setting	ICU	yes	41
		no	66
	Postoperative	yes	22
		no	85
Underlying medical conditions	Ischemic heart disease	yes	26
		no	81
	Acute myocardial infarction	yes	6
		no	101
	Chronic heart failure	yes	24
		no	83
	Diabetes mellitus	no	90
		yes	17
	Sepsis	yes	13
		no	94
	Liver failure	yes	9
		no	98
	Arrhythmia	yes	44
		no	63
	Structural heart disease	yes	9
		no	98
	Hypertension	yes	73
		no	34
Medications	Loop diuretics	yes	65
		no	42
	Antiarrhythmics	yes	34
		no	73
	Antihypertensives	yes	50
		no	57
	Other drugs	yes	106
		no	1

**Table 2:** Descriptive statistics of quantitative parameters of the study population.

	Valid (N)	Mean	SD (σ)	Median	IQR	Minimum	Maximum
Age (y)	107 (100%)	64.2	15.4			17.0	97.0
LVEF (%)	50 (47%)	45.0	17.4			10.0	70.0
SOFA-Score (N)	30 (28%)	9.4	5.0			1.0	20.0
Potassium (mmol/L)	107 (100%)	4.1	0.6			2.3	5.7
Calcium (mmol/L)	74 (70%)	2.4	0.2			1.2	2.8
Magnesium (mmol/L)	80 (75%)	0.9	0.2			0.4	1.5
eGFR (mL/min/1.73m^2^)	107 (100%)	67.5	31.4			4.0	140.0
TSH (mU/L)	70 (65%)	3.6	3.5			0.0	20.1
Initial QTc (ms)	93 (87%)	445.2	25.4			388.0	509.0
QTc under medication (ms)	107 (100%)	497.7	26.4			450.0	570.0
QTc prolongation (ms)	93 (87%)	50.7	29.9			7.0	136.0
KR drugs (N)	107 (100%)			2.0	2.0	0.0	5.0
PR drugs (N)	107 (100%)			1.0	1.0	0.0	3.0
KR + PR (N)	107 (100%)			2.0	2.0	1.0	7.0
MELD-Score (N)	10 (9%)			33.0	10.0	10.0	40.0
HbA1c (%)	20 (19%)			6.2	1.5	4.9	11.4

**Table 3:** Regression coefficient estimates from GLMM for the prediction of absolute QTc under medication.

QTc under Medication	Coefficient	95% Confidence Interval		*p*-Value
Diabetes mellitus	10.652	−0.94	22.245	0.07
Age	−0.348	−0.608	−0.089	0.01
Ischemic cardiomyopathy	14.251	2.126	26.375	0.02
Loop diuretics	6.671	−2.328	15.669	0.14
Initial QTc	0.228	0.038	0.418	0.02
Arrhythmia	9.48	−0.321	19.282	0.06

**Table 4:** Regression coefficient estimates from GLMM for the prediction of QTc prolongation under medication.

QTc prolongation	Coefficient	95% Confidence Interval		*p*-Value
Arrhythmia	20.668	8.892	32.443	<0.001
Antihypertensives	11.596	0.238	22.953	0.045
Age	−0.307	−0.636	0.022	0.067
Potassium	−5.591	−7.806	6.623	0.365

## Data Availability

No publicly available dataset was generated during the study period.

## Supplementary Materials

The following supporting information can be downloaded at: https://www.mdpi.com/article/10.3390/jpm14020172/s1, Table S1: Influence of individual parameters on QTc-time under medication; Table S2: Influence of individual parameters on QTc-prolongation under medication (ΔQTc).
