# Supplementary material for: Developing a Model for Quantifying QTc-Prolongation Risk to Enhance Medication Safety Assessment: A Retrospective Analysis
Source: J Pers Med. 2024 Jan 31;14(2):172. doi: 10.3390/jpm14020172 (PMC10890600; doi:10.3390/jpm14020172)
Supplement: Supplementary file 1 [file jpm-14-00172-s001.zip › jpm-2795479-supplementary.pdf]

Developing a model for quantifying QTc prolongation risk to enhance medication safety assessment: a retrospective analysis

Luis Giovannoni, Gerd A. Kullak-Ublick and Alexander Jetter

Supplementary information

Table S1: Influence of individual parameters on QTc-time under medication.
The selection of tests was based on the nature of the data. Results of both parametric and nonparametric tests are given. Values which were included in the initial modelling are highlighted.

parameter	test	test-value	p-value	test	test-value	p-value
Age (years)	regression	r= -0.143	0.142	Spearman's rank	rho= -0.180	0.063
Sex	t-test	T= 0.973	0.333	Mann-Whitney-U	U= 1158.5	0.278
Hospitalization in the ICU	t-test	T= 0.630	0.530	Mann-Whitney-U	U= 1274	0.613
Postoperative ECG	t-test	T= -0.195	0.846	Mann-Whitney-U	U= 931	0.975
Ischemic heart disease	t-test	T= -1.715	0.089	Mann-Whitney-U	U= 839.5	0.121
Acute myocardial infarction	t-test	T= -1.114	0.268	Mann-Whitney-U	U= 247	0.448
Chronic heart failure	t-test	T= -1.806	0.074	Mann-Whitney-U	U= 765.5	0.085
LVEF (%)	regression	r= -0.141	0.328	Spearman's rank	rho= -0.178	0.217
Diabetes mellitus type II	t-test	T= -2.058	0.042	Mann-Whitney-U	U= 506	0.027
HbA 1c (%)	regression	r= 0.079	0.739	Spearman's rank	rho= -0.059	0.805
Sepsis	t-test	T= 0.684	0.495	Mann-Whitney-U	U= 520.5	0.388
SOFA-score	regression	r= -0.178	0.348	Spearman's rank	rho= -0.139	0.465
Liver failure	t-test	T= 0.942	0.348	Mann-Whitney-U	U= 356	0.340
MELD-score	regression	r= -0.021	0.954	Spearman's rank	rho= -0.422	0.224
Arrhythmia	t-test	T= -1.227	0.223	Mann-Whitney-U	U= 1269.5	0.461
Structural heart disease	t-test	T= -0.602	0.548	Mann-Whitney-U	U= 394	0.598
Hypertension	t-test	T= 0.661	0.510	Mann-Whitney-U	U= 1135	0.478
Potassium (mmol/L)	regression	r= 0.096	0.327	Spearman's rank	rho= 0.064	0.512
Calcium (mmol/L)	regression	r= -0.088	0.462	Spearman's rank	rho= -0.021	0.858
Mg (mmol/L)	regression	r= 0.041	0.715	Spearman's rank	rho= 0.046	0.683
eGFR (ml/min/1.73 m^2)	regression	r= 0.034	0.731	Spearman's rank	rho= 0.046	0.638

TSH (mU/L)	regression	r= 0.000	0.998	Spearman's rank	rho= 0.105	0.385
Loop diuretics	t-test	T= -1.732	0.086	Mann-Whitney-U	U= 1153	0.176
Antiarrhythmics	t-test	T= -2.106	0.038	Mann-Whitney-U	U= 996.5	0.102
Antihypertensives	t-test	T= 0.410	0.682	Mann-Whitney-U	U= 1380	0.779
Initial QTc (msec)	regression	r= 0.291	0.005	Spearman's rank	rho= 0.324	0.002
KR drugs	ANOVA	F= 1.030	0.404	Kruskal-Wallis	H= 4.424	0.490
PR drugs	ANOVA	F= 1.231	0.302	Kruskal-Wallis	H= 3.211	0.360
KR+PR drugs	ANOVA	F= 0.428	0.828	Kruskal-Wallis	H= 2.592	0.763

Table S2: Influence of individual parameters on QTc-prolongation under medication (ΔQTc). The selection of tests was based on the nature of the data. Results of both parametric and nonparametric tests are given. Values which were included in the initial modelling are highlighted.

parameter	test	test-value	p-value	test	test-value	p-value
Age (years)	regression	r= -0.081	0.440	Spearman's rank	rho= -0.108	0.301
Sex	t-test	T= 0.747	0.457	Mann-Whitney-U	U= 896	0.518
Hospitalization in the ICU	t-test	T= 0.039	0.969	Mann-Whitney-U	U= 991	0.629
Postoperative ECG	t-test	T= 1.117	0.267	Mann-Whitney-U	U= 610.5	0.530
Ischemic heart disease	t-test	T= -1.127	0.263	Mann-Whitney-U	U= 658	0.266
Acute myocardial infarction	t-test	T= -0.710	0.479	Mann-Whitney-U	U= 167	0.367
Chronic heart failure	t-test	T= -0.212	0.833	Mann-Whitney-U	U= 732	0.825
LVEF (%)	regression	r= 0.092	0.550	Spearman's rank	rho= 0.048	0.756
Diabetes mellitus type II	t-test	T= 0.253	0.801	Mann-Whitney-U	U= 558.5	0.782
HbA 1c (%)	regression	r= 0.043	0.861	Spearman's rank	rho= 0.210	0.389
Sepsis	t-test	T= 0.039	0.969	Mann-Whitney-U	U= 434	0.551
SOFA-score	regression	r= -0.116	0.548	Spearman's rank	rho= -0.072	0.709
Liver failure	t-test	T= 0.380	0.705	Mann-Whitney-U	U= 364.5	0.861
MELD-score	regression	r= -0.614	0.059	Spearman's rank	rho= -0.817	0.004
Arrhythmia	t-test	T= -2.957	0.004	Mann-Whitney-U	U= 642.5	0.002
Structural heart disease	t-test	T= 1.904	0.060	Mann-Whitney-U	U= 167	0.018
Hypertension	t-test	T= -1.565	0.121	Mann-Whitney-U	U= 781.5	0.282
Potassium (mmol/L)	regression	r= -0.079	0.165	Spearman's rank	rho= 0.005	0.966
Calcium (mmol/L)	regression	r= 0.165	0.182	Spearman's rank	rho= 0.138	0.264
Mg (mmol/L)	regression	r= -0.091	0.439	Spearman's rank	rho= -0.056	0.638
eGFR (ml/min/1.73 m^2)	regression	r= 0.130	0.214	Spearman's rank	rho= 0.105	0.317
TSH (mU/L)	regression	r= -0.110	0.391	Spearman's rank	rho= -0.033	0.798
Loop diuretics	t-test	T= -0.978	0.331	Mann-Whitney-U	U= 886	0.239
Antiarrhythmics	t-test	T= -2.174	0.032	Mann-Whitney-U	U= 587.5	0.005
Antihypertensives	t-test	T= -1.203	0.232	Mann-Whitney-U	U= 969.5	0.404

Initial QTc (msec)	regression	r= -0.609	<0.001	Spearman's rank	rho= -0.585	<0.001
KR drugs	ANOVA	F= 1.291	0.275	Kruskal-Wallis	H= 5.949	0.311
PR drugs	ANOVA	F= 0.288	0.834	Kruskal-Wallis	H= 0.988	0.804
KR+PR drugs	ANOVA	F= 0.929	0.466	Kruskal-Wallis	H= 5.235	0.388
